# Gut and Heart Axis Affects Cardiometabolic Health Through the Ages. A Special Focus on Adolescence

**DOI:** 10.1210/jendso/bvaf213

**Published:** 2026-01-28

**Authors:** Andrea Salzano, Antonio Cittadini

**Affiliations:** Department of Translational Medical Sciences, Federico II University, Via Sergio Pansini 5, Naples 80131, Italy; Interdepartmental Center for Gender Medicine Research “GENESIS,” Federico II University, Via Sergio Pansini 5, Naples 80131, Italy; Cardiology Unit, AORN A Cardarelli, Via Antonio Cardarelli 7, Naples 80131, Italy; Department of Translational Medical Sciences, Federico II University, Via Sergio Pansini 5, Naples 80131, Italy; Interdepartmental Center for Gender Medicine Research “GENESIS,” Federico II University, Via Sergio Pansini 5, Naples 80131, Italy

**Keywords:** TMAO, metabolites, cardiovascular diseases, adolescence, cardiometabolic health, gut

The research field recently strongly pointed out the gastrointestinal system (GI) as an innovative model of interest in cardiovascular disease (CVD) pathophysiology (ie, the “gut hypothesis”) [1]. The host-microbiota interaction has been widely explored, suggesting not only an association between CVD (eg, ischemic heart diseases, heart failure) and gut impairment, but also a causative role [1]. Going further is the naive definition of gut dysfunction as a “simple” intestinal disorder, with inflammation and oxidative stress deriving from the gut-heart axis impairment currently considered as key players in the pathogenesis of CVD, consequences of a gut barrier dysfunction, often caused by wall ischemia and/or congestion [1]. Two principal mechanisms have been suggested: First, the gut-derived metabolites directly affect the host, entering in the general circulation—“metabolism-dependent” effects (proatherogenic effects and proinflammatory effects); and second, specific bacterial components (lipopolysaccharides) translocate in the systemic circulation, primarily inducing and subsequently chronically promoting an inflammatory state—“metabolism-independent” effects. In addition, 10 bacterial taxa have been recently associated with a higher incidence of adverse CV events in patients with coronary artery disease, further supporting the role of microbiota in development of CVDs [2].

Among the microbiota-dependent metabolites, trimethylamine *N*-oxide (TMAO), an intestinal microbiota-dependent product of dietary carnitine and choline [1], has been shown to drive this association. Specifically, TMAO derives from the metabolism of phosphatidylcholine or L-carnitine into trimethylamine by bacteria and the subsequent conversion into TMAO in the liver by flavin-containing monooxygenases; red meat and eggs being rich in these substrates. TMAO represents a putative link between CVDs and the Western diet [1].

In this context, with the aim of investigating a possible association between TMAO and clinical, metabolic, and inflammatory variables in adolescents, Iorra et al [3] performed a multicenter cross-sectional analysis from the Study of Cardiovascular Risks in Adolescents (Portuguese acronym ERICA), a nationwide, school-based study conducted in Brazil between 2013 and 2014 designed to assess the prevalence of metabolic syndrome and CV risk factors in adolescents. For this purpose, 4446 individuals aged 12 to 17 years, attending schools in all 27 Brazilian federation units, were evaluated through questionnaires and clinical and anthropometric assessments. Considering the possible relationship between TMAO levels and inflammatory state, individuals with C-reactive protein (CRP) above 10 mg/L were excluded. The associations between TMAO levels and cardiometabolic aspects were investigated using 3 models adjusted for different covariates. Specifically, sex, age, skin color, and socioeconomic status (first model); creatinine and percentage of total energy intake derived from ultra-processed foods to account for dietary factors (second model); and the second model plus body mass index (BMI; third model). In addition, the effect of prior diagnosis or treatment for diabetes on fasting glucose, glycated hemoglobin A_1c_, and insulin levels were evaluated and a comparative analysis was performed of TMAO levels between patients with self-reported comorbidities (hypertension, diabetes, and dyslipidemia) and those without these conditions.

As a result, in all 3 models, TMAO levels were positively associated with waist circumference (WC) and BMI *Z* score. In addition, the higher tertiles of TMAO were positively associated with CRP levels. On the other hand, a negative association between fasting plasma glucose was observed. Finally, no differences were reported about TMAO levels between self-reported comorbidities and healthy adolescents [3].

The present study supports the role of the gut in the development of metabolic and CV risk, adding to the topic the investigation of adolescents; indeed, available data at present have focused on healthy adults or older individuals, or on CVD patients. To date, only a few studies have investigated the possible association of TMAO with metabolic health and CVD in adolescents, with inconclusive results [3-5]. In the present study, TMAO levels are associated, even when adjusted for several confounders, with WC and BMI, indicating a link between TMAO and metabolic syndrome and obesity, 2 well-known modifiable risk factors for CVD; this is in line with results from a different study, in which TMAO levels not only showed a positive correlations to BMI, WC, and waist-to-height ratio, but were also predictive for these parameters [5]. Regarding fasting plasma glucose, in the present investigation prevalence of diabetes mellitus (DM) is very low (3.2%), with (presumably) all patients affected by type 1. The existing literature on the topic is based on type 2 DM, with only scarce data on type 1 [6], and TMAO levels have been indicated as a possible index of hepatic insulin resistance [6]. Conflicting results are available; a dose-dependent association with TMAO levels have been advocated as a possible risk factor for future risk of DM, and with DM able to magnify the association between TMAO and CVD [6]; on the other hand, a negative association between higher TMAO levels and a lower risk of future DM has already been shown, with a very high variability linked to sex, in particular in healthy individuals [6].

Finally, in the present investigation, TMAO levels were associated with CRP levels, indicating a connection between TMAO levels and low-grade systemic inflammation, a further risk factor for CVD. Surprisingly, no differences were reported between participants with comorbidities and healthy adolescents; however, the number of the first group was very low, not allowing for a proper comparison [3]. Notably, in the Growing Up in Australia's Child Health CheckPoint Study [4], TMAO precursors (ie, choline, betaine, carnitine) but not TMAO itself were associated with adverse cardiometabolic and inflammatory phenotypes in enrolled children and adults. However, on the one hand ethnic and geographic differences have been described, explaining this possible discrepancy between the studies [7, 8]. On the other hand, it has been shown that other metabolites of the choline/carnitine-TMA-TMAO pathway are involved in the development of CVD [1].

In conclusion, in the context of the “gut-heart axis” model, the present article further supports the association between TMAO and metabolic and CVDs; specifically, the present study adds to the knowledge focusing on adolescents, showing an association between TMAO levels and risk factors for CVD development (ie, WC, obesity, and inflammation).

## Abbreviations

BMI: body mass index
CRP: C-reactive protein
CVD: cardiovascular disease
DM: diabetes mellitus
TMAO: trimethylamine *N*-oxide
WC: waist circumference
